# Continuous anesthesia for 60 days in an isosmotic environment does not impair limb or cardiac regeneration in the axolotl

**DOI:** 10.1038/s41598-023-42339-z

**Published:** 2023-09-11

**Authors:** Sofie Amalie Andersson, Anita Dittrich, Henrik Lauridsen

**Affiliations:** Department of Clinical Medicine, Aarhus University, Palle Juul-Jensens Boulevard 11, 8200 Aarhus N, Denmark

**Keywords:** Cardiac regeneration, Self-renewal, Animal physiology

## Abstract

Longitudinal animal experiments in the field of regenerative biology often require repeated use of short-term anesthesia (minutes to a few hours). Regain of consciousness limits the level of acceptable invasiveness of procedures, and it makes it difficult to untangle behavioral changes caused by injury to physiological processes involved in the regenerative response. Therefore, a method to keep a regenerative research animal in a comatose state under continuous anesthesia during regenerative experiments often spanning months, would be ethically and experimentally desirable. Here we report on a method using propofol based anesthesia in an isosmotic environment that allows for continuous anesthesia of regenerating axolotls for 60 days with a 75% survival rate, thus spanning the majority of a full regenerative cycle following limb amputation or cryoinjury to the heart. No differences were detected in the axolotl’s ability to regenerate amputated limbs and cardiac cryo-injury while anesthetized, however some regenerative failures in the limb were observed in both anesthetized and unanesthetized control groups, most likely caused by prolonged fasting. Sixty days of anesthesia may be approaching a level were kidney function is affected, but the 75% surviving anesthetized animals recovered well after anesthesia and showed a full behavioral recovery within 17 days.

## Introduction

The ability to regenerate lost or damaged tissue would be of great benefit for people suffering from the loss of bodily functions, such as organ failure or amputated limbs. However, adult mammals, humans included, have rather limited regenerative potential compared with other species such as some teleost fish and urodele salamanders^1–4^. One popular model organism capable of intrinsic tissue regeneration is the Mexican axolotl *Ambystoma mexicanum* (Shaw and Nodder, 1798), which is able to regenerate a wide range of organs including parts of the brain^5^, limbs^6^, tail^7^, spinal cord^8^, lungs^9^, as well as the heart^10–12^.

Tissue injury is a fundamental part of regenerative research. From an ethical viewpoint, injury to research animals must be minimized as far as experimentally possible, and procedures for proper anesthesia and analgesia are a cornerstone in modern animal research. From an experimental viewpoint, adverse behavioral effects of injury can confound physiological measurements. Continuous anesthesia in a regenerative study would represent a method to potentially uncouple behavior from the regenerative response. The longest anesthesia period previously achieved in a regeneration competent species was 2 days in zebrafish^13^, and 7 days in the axolotl^14^. While the zebrafish needs intubation to survive extended anesthesia, the axolotl with external gills and cutaneous gas exchange can be ventilated using bubbling of the housing medium^14^, which represents a simpler setup and thus a more approachable system for a long-term anesthesia study spanning the majority of a regenerative cycle.

It has previously been demonstrated that propofol (2,6-diisopropylphenol) is applicable as an immersion anesthetic for axolotls^15^, and that in some cases propofol is preferable to more commonly used amphibian anesthetics such as MS-222 (ethyl 3-aminobenzoate methanesulfonic acid) and benzocaine (ethyl 4-aminobenzoate) as it requires no use of organic solvents for solubility nor buffering to adjust pH, both of which may cause skin irritation or lesions with prolonged exposure^16^. Propofol is a proven hypnotic^17^ (i.e. sleep-inducing) and it does not affect cardiac function in axolotls significantly. Furthermore, repeated but intermittent exposures to propofol does not affect gross limb regeneration^15^. Undiluted propofol should be handled with care and proper personal protection equipment e.g. nitrile gloves and glasses should be used.

In this study, we set out to develop a method using propofol anesthesia to continuously keep axolotls in a comatose state for 60 days, thus spanning the majority of the regeneration cycle of both the heart and the limb. We did this in steps using three pilot experiments informing a main experiment to confirm that the anesthesia protocol was adequate and modifications to the housing medium could be integrated with regenerative experiments. In specific, we designed a series of experiments to test the following six hypotheses:

*H1: Healthy axolotls can be continuously anesthetized for 60 days in conventional hyposmotic housing medium without adverse effects.*

*H2: Osmolarity of housing medium affects swelling in continuously anesthetized healthy axolotls.*

*H3: Unanesthetized axolotls can perform heart regeneration in an isosmotic housing medium.*

*H4: Unanesthetized and fasting axolotls can perform heart regeneration in both hyposmotic and isosmotic housing medium.*

*H5: Continuously anesthetized axolotls in an appropriate housing medium can perform heart and limb regeneration at a similar rate as unanesthetized control animals over the course of 60 days.*

*H6: Heart and limb regeneration constitutes a metabolic challenge significantly affecting oxygen consumption rate when adjusting for behavioral changes following injury.*

## Results

### Waste production, propofol consumption and the effect of long term anesthesia in tap water

**Figure 1:**
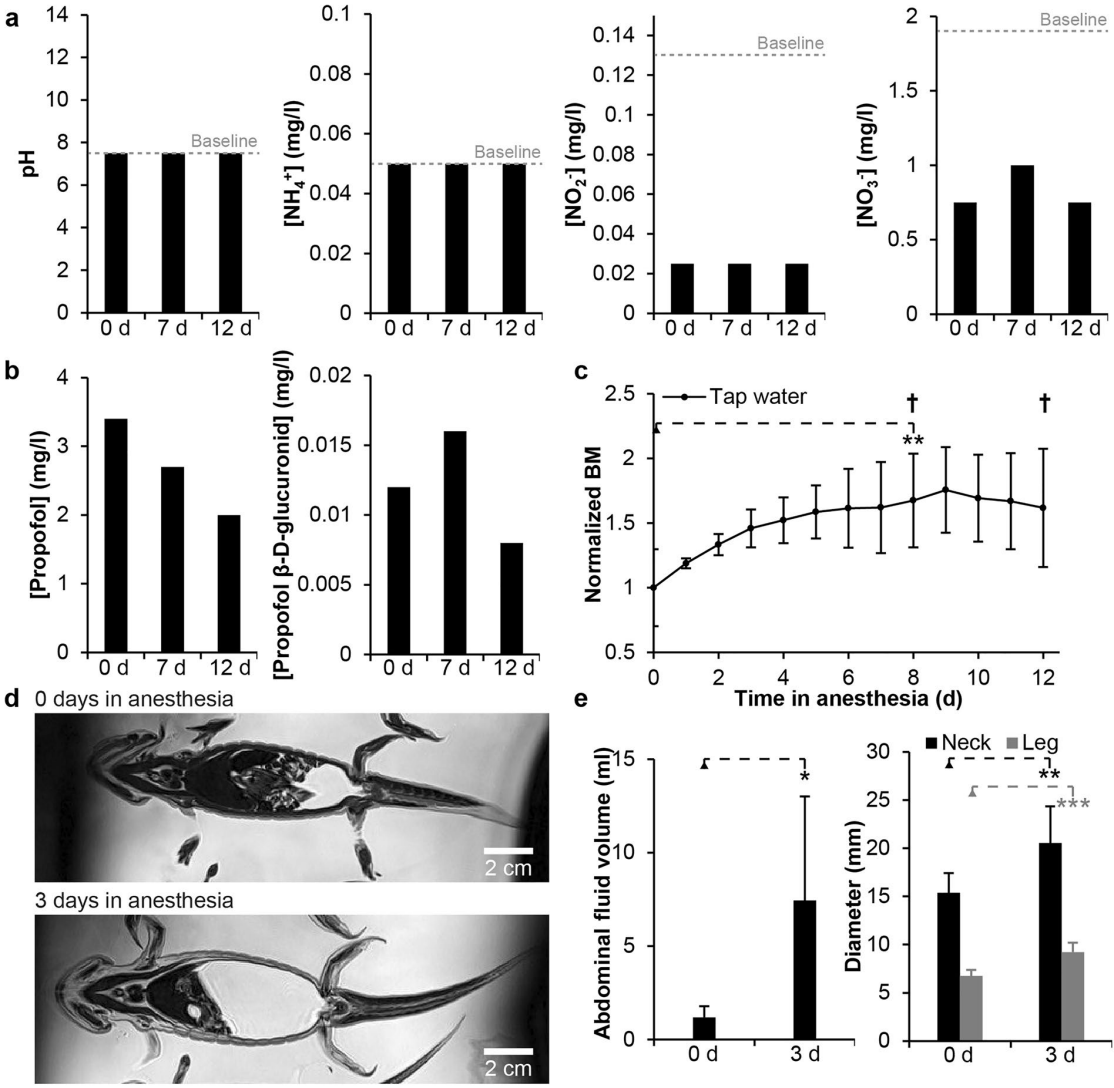
Waste production, propofol consumption and long-term anesthesia in conventional tap water housing medium. (**a**) pH, ammonium ion, nitrite ion and nitrate ion concentration over time in 10 l housing medium containing six axolotls during 12 days of continuous anesthesia. (**b**) Propofol and propofol β-d-glucuronid concentration over time in housing medium. (**c**) Normalized body mass (BM) over time. Body mass was significantly increased (**p < 0.01, based on paired t-test; n = 6) until an animal died at day 8 and again at day 12 (cross symbol) and anesthesia was ended. (**d**) Representative virtual coronal magnetic resonance imaging slices of the same axolotl at the beginning of anesthesia and after 3 days showing gradual built up of abdominal fluid. (**e**) Significant increase in abdominal fluid (*p < 0.05, based on paired t-test; n = 6) and diameter at the level of the neck (**p < 0.01, based on paired t-test; n = 6) and hind legs (***p < 0.001, based on paired t-test; n = 6) following 3 days in propofol anesthesia in tap water housing medium.

The six animals of the Pilot 1 experiment were housed together in a total of 10 l of tap water. None of the measured waste parameters were higher for the 12 day duration of the experiment than at baseline level (Fig. 1a). pH level and ammonium ion concentration were constant throughout the experiment at 7.5 and 0.05 mg/l, respectively, whereas nitrite and nitrate ion concentrations were much lower during fasting anesthesia than at regular housing baseline levels (Fig. 1a). Propofol concentration in housing water decreased as inflow rate was lowered to keep the animals under anesthesia at the minimum-required dose and there was no build-up of the propofol metabolite propofol β-d-glucuronid (Fig. 1b). Body mass was significantly increased to a maximum of 1.7 fold at day 8 of the experiment (paired t-test day 0 vs. day 8, n = 6, p = 0.0026) where the first animal died in anesthesia (Fig. 1c). Magnetic resonance imaging revealed that body mass increase was due to both a variable but significant build-up of abdominal fluid (paired t-test day 0 vs. day 3, n = 6, p = 0.049) and general edema of soft tissues exemplified at the neck (paired t-test day 0 vs. day 3, n = 6, p = 0.0012) and leg (paired t-test day 0 vs. day 3, n = 6, p = 0.00017) (Fig. 1d,e). Hematocrit was reduced from 30.3 ± 7.4 to 23.8 ± 5.3%, although not significantly so (paired t-test day 0 vs. day 3, n = 6, p = 0.19), but plasma osmolarity was significantly reduced from 208 ± 8.1 mOsm/l to 182.3 ± 6.3 mOsm/l (paired t-test day 0 vs. day 3, n = 6, p = 0.00052). In addition to the animal dying in anesthesia at day 8, another animal died at day 12, and anesthesia was stopped. The remaining four animals recovered well and were back to 99% of pre anesthesia body mass within 7 days (paired t-test day 0 vs. recovery day 7 (19 days since start of anesthesia), n = 4, p = 0.74).

### Short-term anesthesia and heart regeneration in isosmotic housing medium

**Figure 2:**
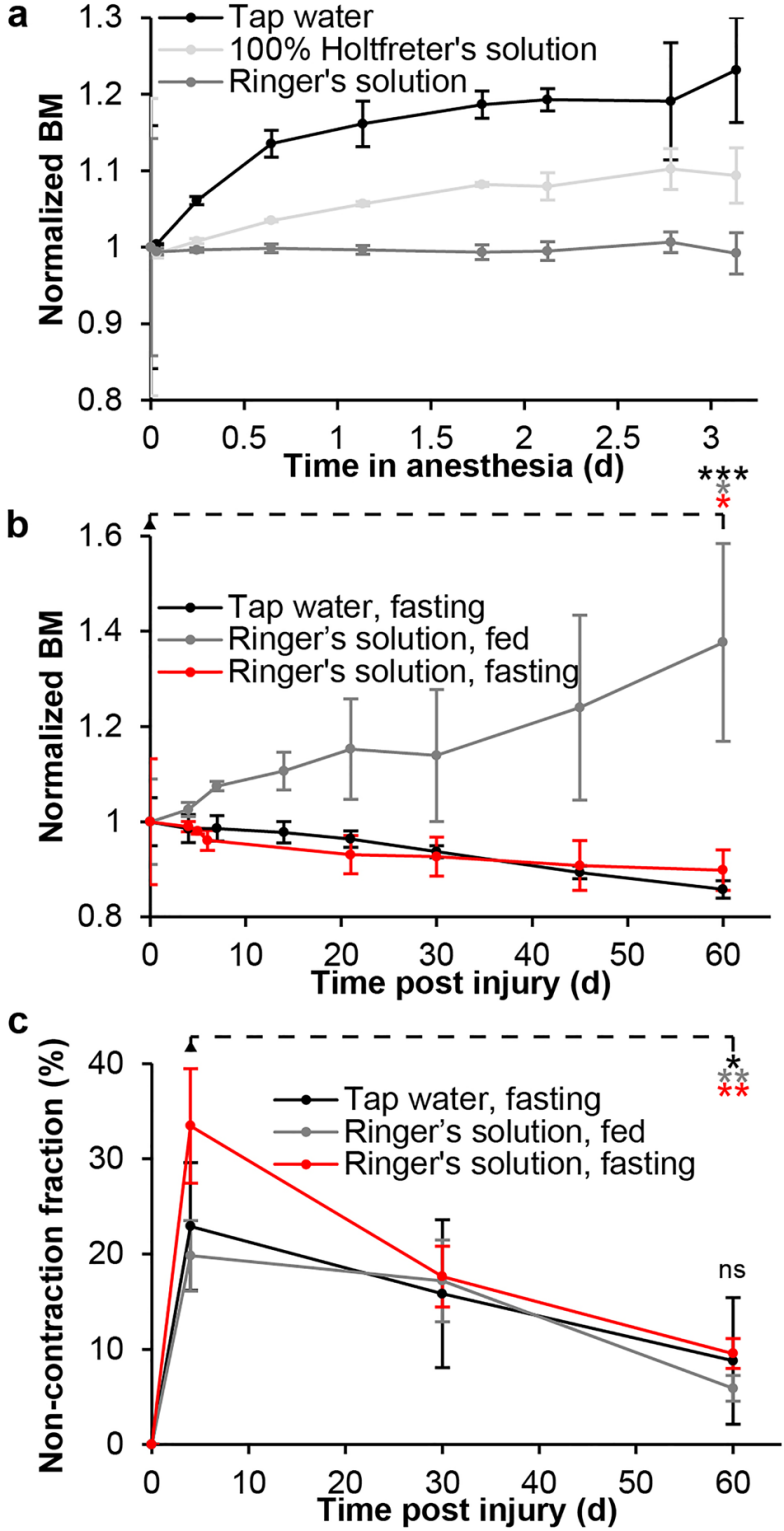
The effect of regulating osmolarity in housing medium and of fasting on body mass over time and cardiac regeneration. (**a**) Normalized body mass (BM) over the course of 3 days of propofol anesthetized axolotls (three animals per group) housed in hyposmotic tap water, hyposmotic 100% Holtfreter’s solution or isosmotic axolotl adjusted Ringer’s solution. (**b**) Normalized body mass of conscious axolotls with a cardiac cryoinfarction housed in either tap water or Ringer’s solution with or without feeding for 60 days. Axolotls housed in Ringer’s solution with feeding significantly increased their body mass over the course of the experiment (*p < 0.05, based on paired t-test; n = 4), whereas fasted animals housed in both tap water and Ringer’s solution significantly decreased their body mass over time (***p < 0.001, based on paired t-test; n = 4 and *p < 0.05, based on paired t-test; n = 4, respectively). (**c**) The non-contraction fraction of the ventricle in axolotls with a cardiac cryoinfarction housed in either tap water or Ringer’s solution with or without feeding for 60 days. All groups showed functional regeneration of the heart expressed as a decrease in the non-contraction fraction (Tap water, fasting: *p < 0.05, Ringer’s solution, fed: **p < 0.01, Ringer’s solution fasted: **p < 0.01, respectively, all based on paired t-tests; n = 4) and there was no significant difference in non-contraction fraction between groups (*ns* not significant, based on one-way ANOVA; n = 4 pr. group).

Housing anesthetized axolotls of the Pilot 2 experiment in both tap water (14.0 mOsm/l) and 100% Holtfreter’s solution (127.2 mOsm/l), both of which are hyposmotic to axolotl plasma (208 mOsm/l), resulted in swelling and body mass increase as observed in the Pilot 1 experiment (Fig. 2a). This was contrasted by housing in isosmotic axolotl Ringer’s solution where body mass was stable (Fig. 2a). To our knowledge, long-term usage of isosmotic housing medium has never been reported for the axolotl, thus we needed to establish whether conscious axolotls survive well in this medium and are able to regenerate tissue before attempting to use this medium in a long-term continuous anesthesia setup. Additionally, since anesthetized axolotls are not able to feed, we needed to establish whether prolonged fasting affected the ability to regenerate in both conventional tap water housing medium as well as in isosmotic housing medium. In the Pilot 3 experiment, axolotls housed in tap water and isosmotic Ringer’s solution survived equally well. A significant reduction in body mass was observed for both fasting animals housed in tap water (a body mass reduction to 86% of baseline level, paired t-test day 0 vs. day 60, n = 4, p = 0.00029) and Ringer’s solution (a body mass reduction to 90% of baseline level, paired t-test day 0 vs. day 60, n = 4, p = 0.020) whereas fed animals housed in Ringer’s solution experienced a significant increase in body mass (a body mass increase to 138% of baseline level, paired t-test day 0 vs. day 60, n = 4, p = 0.049) (Fig. 2b). All groups, irrespective of housing medium and feeding state, were able to muster a regenerative response to cryoinjury to the heart as observed by a significant decrease of the non-contraction fraction of the myocardium from day 4 where the injury was maximal to day 60 (tap water and fasting: paired t-test day 0 vs. day 60, n = 4, p = 0.016; Ringer’s solution and fed: paired t-test day 0 vs. day 60, n = 4, p = 0.00050; Ringer’s solution and fasting: paired t-test day 0 vs. day 60, n = 4, p = 0.0023) (Fig. 2c). There was no significant difference between non-contraction fraction between groups at day 60 (one-way ANOVA, F(2,9) = 0.94, p = 0.43).

### Long-term anesthesia in isosmotic housing medium

**Figure 3:**
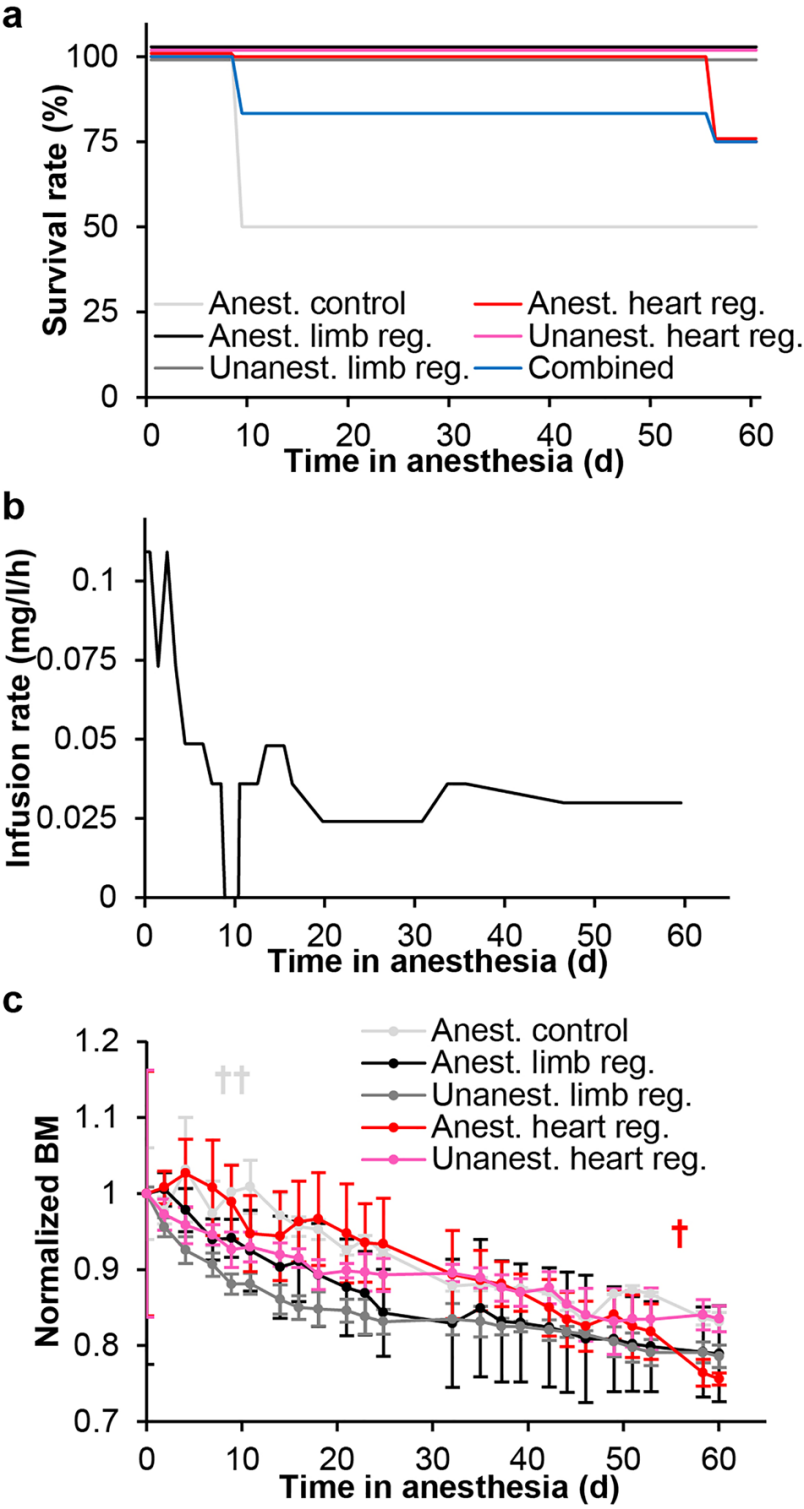
Survival rate, propofol infusion rate and body mass over time in continuously anesthetized axolotls in isosmotic housing medium. (**a**) Survival rate over time in anesthetized control axolotls (Anest. control) without injury, anesthetized and unanesthetized axolotls performing limb regeneration (Anest. limb reg. and Unanest. limb reg.), anesthetized and unanesthetized axolotls performing heart regeneration (Anest. heart reg. and Unanest. heart reg.), and combined for all anesthetized axolotls. (**b**) Propofol infusion rate over the course of 60 day. After the loss of two anesthetized control animals at day 9, infusion was briefly stopped to allow the animals to metabolize some of the built up propofol in the tissue while still being under complete anesthesia, before propofol infusion was resumed. (**c**) Normalized body mass over time of all groups. The loss of two anesthetized control animals at day 9 and one anesthetized heart regeneration animal at day 56 are indicated by cross symbols.

Overall survival rate in the main experiment of long term anesthesia during heart and limb regeneration was 75% (Fig. 3a). Included in this is the loss of two anesthetized but uninjured control animals at day 9 of the experiment. Propofol infusion rate was gradually lowered during the first 20 days of the experiment with a complete infusion stop for 2 days between day 8.5 and day 10.5 to readjust anesthesia to a level where the animals were kept unconscious at the minimum-required dose to reduce the risk of oversedation (Fig. 3b). Body mass steadily decreased for anesthetized as well as fasting unanesthetized animals (Fig. 3c). Uncorrected multiple student’s t-tests showed significant differences in normalized body mass at day 60 for the heart regeneration groups (p = 0.012) and at day 2 and 9 for the limb regeneration groups (p = 0.025 and p = 0.019, respectively). However, when applying Bonferroni correction to the significance level to account for multiple comparisons no significant difference in normalized body mass between intervention groups (anesthetized heart regeneration and anesthetized limb regeneration) and their relative control groups (unanesthetized heart regeneration and unanesthetized limb regeneration) where found throughout the experiment.

### Heart and limb regeneration during long-term anesthesia

**Figure 4:**
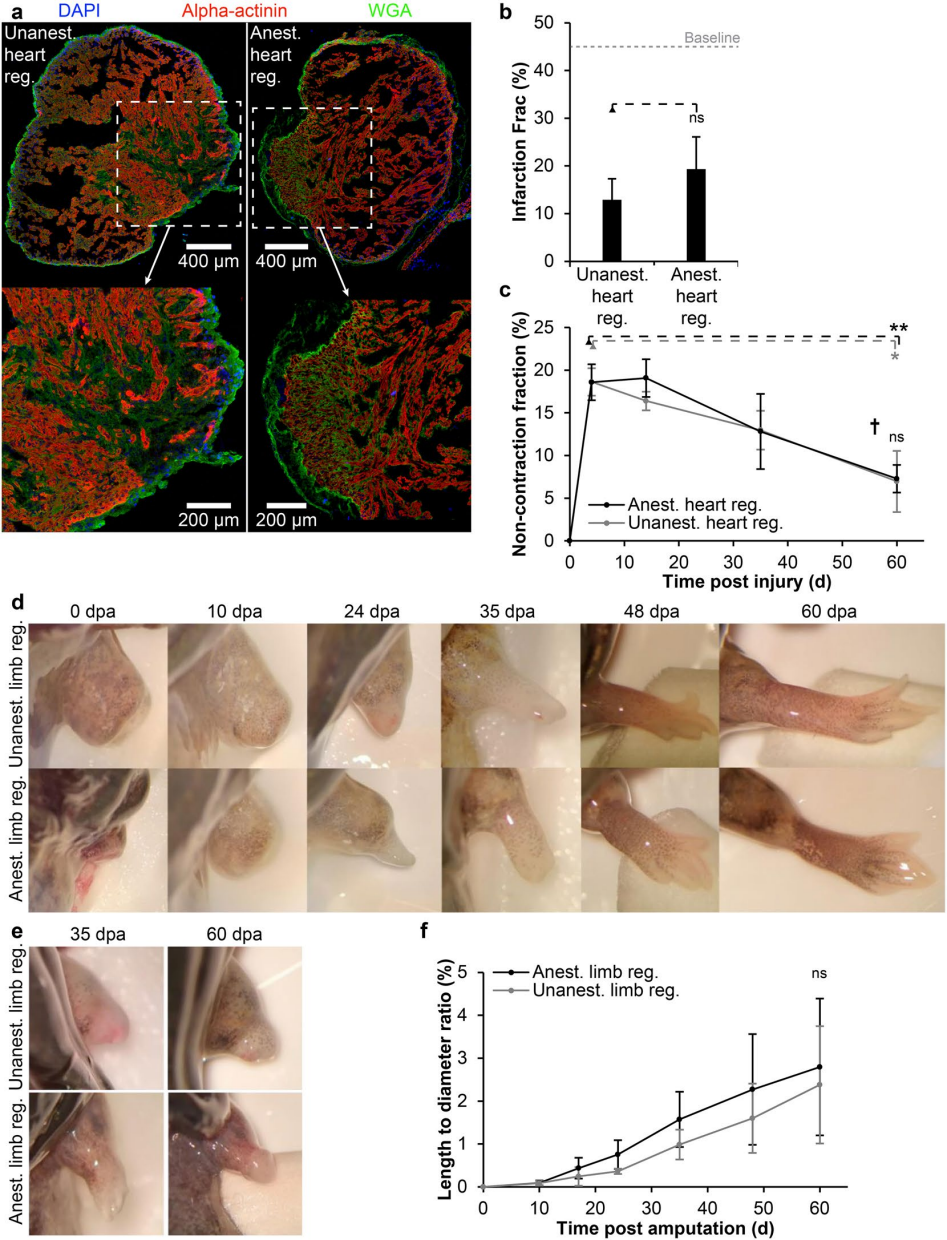
Heart and limb regeneration during continuous propofol anesthesia. (**a**) Representative cryosections of the cardiac infarction zone in an unanesthetized (left) and an anesthetized axolotl (right) after 60 days of regeneration. The injury zone is still visible via wheat-germ agglutinin staining in both hearts at this time point but it is highly infiltrated by cardiomyocytes made visible by alpha-actinin staining. White boxes are magnified ×2 in images at the bottom. (**b**) There was no significant difference in infarction fraction measured by quantitative histology between hearts at 60 days post injury in the unanesthetized (Unanest. heart reg) and the anesthetized group (Anest. heart reg) (ns, not significant, based on unpaired t-test, n = 3 for each group). (**c**) The non-contraction fraction of the ventricle in unanesthetized and anesthetized axolotls with a cardiac cryoinfarction over the course of 60 days. Both groups showed a significant decrease in non-contraction fraction from day 4 to day 60 post injury (*p < 0.05 and **p < 0.01, respectively, based on paired t-tests; n = 3 for each group) and there was no significant difference between unanesthetized and anesthetized animals at day 60 (*ns* not significant, based on unpaired t-test; n = 3 for each group). (**d**,**e**) Representative photos of amputated right front limb in anesthetized and unanesthetized axolotls at 0, 10, 24, 35, 48 and 60 days post amputation (dpa). Most axolotls showed complete limb regeneration except from one animal from each of the anesthetized and unanesthetized groups showing a regression of the initially regenerating limb (**e**). (**f**) Length to diameter ratio of regenerating limbs over time. There was no significant difference of limb size at day 60 between anesthetized and unanesthetized axolotls (unpaired t-test, n = 4 and 3, p = 0.77).

Quantitative histology of harvested hearts at day 60 revealed no significant difference in infarction fraction of the anesthetized or the unanesthetized heart regeneration groups (unpaired t-test, n = 3, p = 0.33) (Fig. 4a,b). Similarly, echocardiography showed no significant difference in non-contraction fraction between the anesthetized and the unanesthetized heart regeneration groups at day 60 (two-way ANOVA with repeated measures, F(1,4) = 0.099, p = 0.77) (Fig. 4c). Both groups showed a significant decrease in non-contraction fraction at day 60 compared to day 4 where the size of the non-contracting portion of the ventricle was maximal (two-way ANOVA with repeated measures, F(1,4) = 0.099, p = 0.0022 and paired t-tests for day 4 vs. day 60: anesthetized heart regeneration, n = 3, p = 0.0027 and unanesthetized heart regeneration, n = 3, p = 0.014).

In the anesthetized limb regeneration group, three out of four animals performed complete limb regeneration within 60 days (Fig. 4d). Likewise, two out of three animals in the unanesthetized control group performed complete limb regeneration (Fig. 4d). The remaining animals from both groups both developed a blastema and a palette, but instead of completing the development, the process regressed (Fig. 4e). There was no significant difference in the length to diameter ratio of limbs between the anesthetized and unanesthetized groups (unpaired t-test, n = 4 and 3, p = 0.77) (Fig. 4f).

### Oxygen consumption rate during long-term anesthesia and regeneration

**Figure 5:**
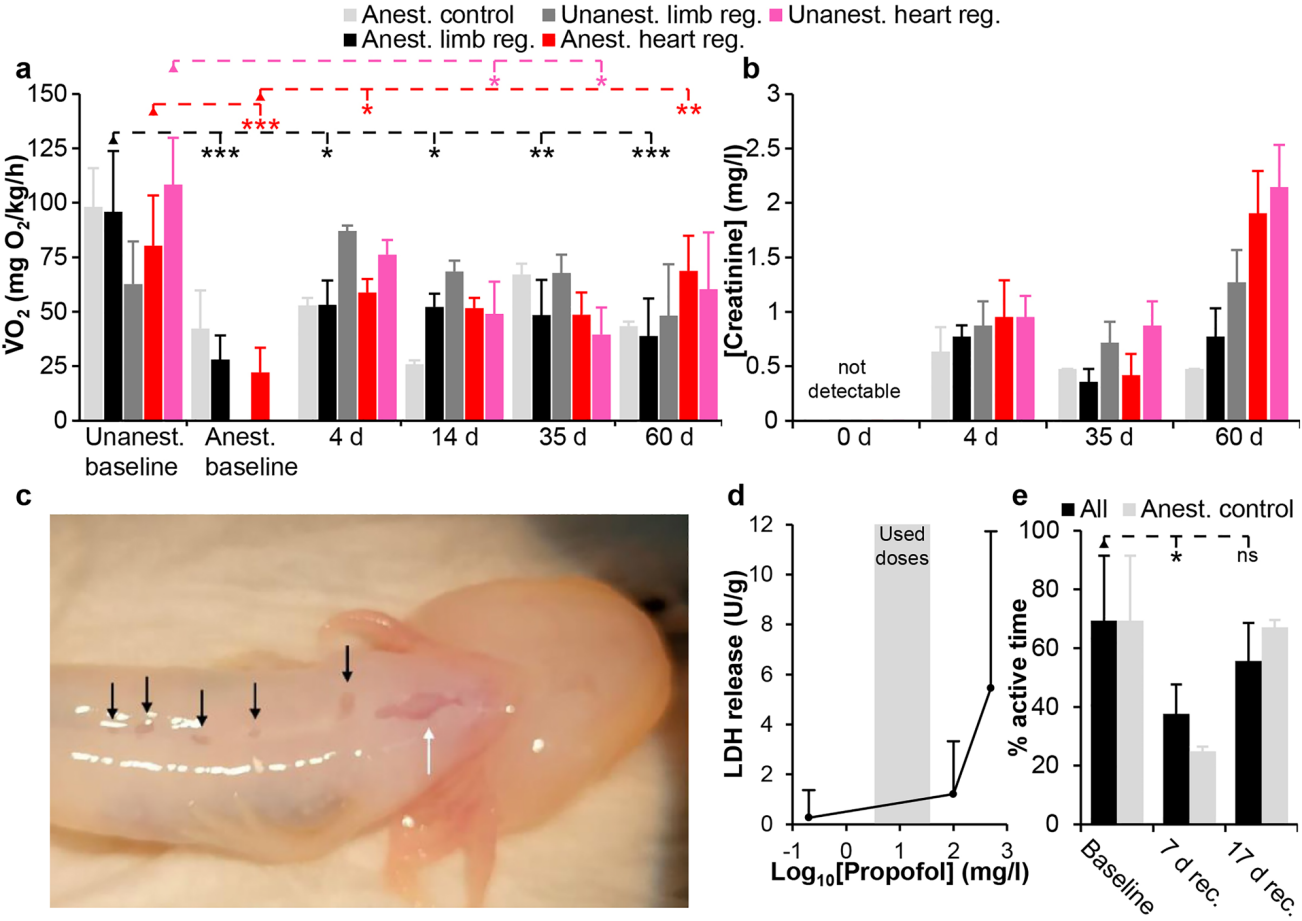
Oxygen consumption, skin toxicity and behavioral effects of continuous propofol anesthesia. (**a**) Oxygen consumption rate over time in anesthetized control axolotls (Anest. control) without injury, anesthetized and unanesthetized axolotls performing limb regeneration (Anest. limb reg. and Unanest. limb reg.), anesthetized and unanesthetized axolotls performing heart regeneration (Anest. heart reg. and Unanest. heart reg.). Oxygen consumption rate was significantly different from unanesthetized baseline at some time points in the anesthetized limb regeneration group and in both the anesthetized and the unanesthetized heart regeneration groups (see text for details). (**b**) plasma creatinine level in the different treatment groups over time. Although not significantly regulated, there was a tendency of an increase in creatinine level over time. (**c**) Small skin lesions were visible on the ventral surface of some anesthetized axolotls (black arrows). White arrow points to healing skin at the heart injury zone. (**d**) Lactate dehydrogenase (LDH) release from skin samples showed increased toxicity of propofol at high concentrations although not pronounced within the used therapeutic zone. (**e**) Percent time of axolotls being active at baseline and again at 7 days of recovery and 17 days of recovery following 60 days of anesthesia. Data is shown for both previously anesthetized control animals as well as these animals in combination with previously anesthetized limb regeneration animals (All). Long term propofol anesthesia significantly affected activity levels 7 days after recovery (p = 0.014), whereas at 17 days post recovery it was normalized to pre anesthesia level (p = 0.35).

One-way ANOVA with repeated measures was performed to detect significant changes in oxygen consumption rate for unanesthetized as well as anesthetized animals regenerating either a limb or a cardiac injury. For unanesthetized limb regeneration, no significant change in oxygen consumption rate was detected, F(4,8) = 2.907, p = 0.093 (Fig. 5a). For unanesthetized heart regeneration, however, one-way ANOVA with repeated measures revealed a significant change in oxygen consumption rate over time, F(4,8) = 5.078, p = 0.025. Tukey HSD post hoc test showed a significant decrease in oxygen consumption rate at 14 dpi (p = 0.046) and 35 dpi (0.022) relative to baseline oxygen consumption rate (Fig. 5a). For anesthetized limb regeneration, one-way ANOVA with repeated measures revealed significant changes in oxygen consumption rate over time F(5,15) = 9.39, p = 0.00033 and Tukey HSD post hoc test showed that awake baseline oxygen consumption was significantly higher than both anesthetized baseline (p = 0.00016), day 4 (p = 0.012), day 14 (p = 0.010), day 35 (p = 0.0052), and day 60 (p = 0.00097) post injury (Fig. 5a). For anesthetized heart regeneration, one-way ANOVA with repeated measures revealed significant changes in oxygen consumption rate over time F(5,15) = 6.89, p = 0.0016), and Tukey HSD post hoc test showed the difference to be between anesthetized baseline and day 4 (p = 0.036), day 60 (p = 0.0062) post injury as well as awake baseline (p = 0.00081) (Fig. 5a).

### Renal function during long-term anesthesia and regeneration

Plasma creatinine levels prior to injury and propofol induced long-term anesthesia were below the detection limit of the assay. One-way ANOVA with repeated measures for the different treatment groups did not reveal significant changes in creatinine levels (unanesthetized limb regeneration: F(2,4) = 1.90, p = 0.26; unanesthetized heart regeneration: F(2,4) = 7.18, p = 0.050; anesthetized limb regeneration: F(2,6) = 1.90, p = 0.229; anesthetized heart regeneration: F(2,4) = 6.26, p = 0.059), but there was a tendency of increased levels of plasma creatinine a day 60 post injury indicating reduced kidney function (Fig. 5b).

### Propofol toxicity to axolotl skin

Small skin lesions were observed on 25% of the axolotls during long-term anesthesia (Fig. 5c). These were first observed after 20 days of anesthesia and appeared as localized thinning of the skin, but with no signs of infection. Analyzing propofol toxicity on previously unexposed skin samples revealed increased lactate dehydrogenase release indicating cellular toxicity at high supratherapeutic concentrations of propofol (Fig. 5d). Within the therapeutic window used in the long-term anesthesia setup LDH release was limited (Fig. 5d).

### Behavioral effect of long-term anesthesia

Originally, the analysis of a potential behavioral effect of long-term anesthesia was intentionally conducted in a paired setup using the anesthetized but uninjured control group to avoid confounding behavioral responses to injury. However, since two out of four animals in this group died 9 days into the experiment, rendering the remaining group of two animals too small to conduct statistical tests, we instead used the remaining two control animals in combination with the four anesthetized limb regeneration animals in follow up measurements of active time at seven and 17 days after ending anesthesia. Long term propofol anesthesia significantly affected activity levels (ANOVA, F(2,13) = 5.82, p = 0.016), but post hoc Tukey HSD revealed that the percentage of active time was only significantly decreased 7 days after recovery (p = 0.014), whereas at 17 days post recovery it was normalized to pre anesthesia level (p = 0.35) (Fig. 5e).

## Discussion

Abiotic factors in the housing medium are important to consider when housing aquatic species like the axolotl. Our early experiments in long-term anesthesia showed that a gradual buildup of nitrogenous waste products was not a concern within the 1 week period between regular water changes. On the contrary, the fasting state of the animals caused a steady state of abiotic parameters or even a decrease in nitrate and nitrite ion levels (Fig. 1a). Similarly, the used propofol infusion rate did not result in a gradual increase in concentration of either propofol or the metabolite propofol β-d-glucuronid (Fig. 1b), demonstrating that the proposed anesthesia protocol yielded an acceptable abiotic environment. However, housing in the tap water medium conventionally used in our lab and elsewhere (locations with chemically treated water may use purified water with added salts to achieve 40% Holtfreter’s solution^18^) was not compatible with long term anesthesia due to animal swelling, thus hypothesis 1, that healthy axolotls can be continuously anesthetized for 60 days in conventional hyposmotic housing medium without adverse effects could be rejected (Fig. 1c–e). The underlying mechanisms of swelling are not clear but are most likely associated with a disruption of water homeostasis during continuous anesthesia causing a significant decrease in plasma osmolarity and gradually impaired kidney function. However, as demonstrated in subsequent experiments, housing axolotls in isosmotic housing medium alleviates this problem and does not impair regenerative capacity thus confirming hypothesis 2 that the osmolarity of housing medium effects swelling in continuously anesthetized healthy axolotls as well as hypothesis 3, that unanesthetized axolotls can perform heart regeneration in an isosmotic housing medium (Fig. 2). The axolotl is generally not considered a brackish water tolerant species^19^, although a single report does mention the axolotl (specifically *Ambystoma mexicanum*) to inhabit the brackish Mexican Laguna Alchichica with a salinity of 8.3–9 g/l^20^, i.e. similar to the salinity of axolotl body fluids. However, this is most likely a confusion with another neotenic member of the tiger salamander complex *Ambystoma taylori* Brandon, Maruska and Rumph 1982, a well-known inhabitant of this brackish crater lake^21,22^. Nevertheless, our study demonstrates that also the axolotl can tolerate prolonged housing in isosmotic medium, which is experimentally useful in situations where water homeostasis cannot be maintained by the animal itself.

Anesthetized animals were not able to eat during anesthesia, so in order to compare the regenerative processes of anesthetized and unanesthetized axolotls, all animals were fasted for the duration of the experiment. To avoid potential gastrointestinal problems associated with anesthesia, all animals were additionally fasted for 14 days prior to the start of the experiment^23^. As such, the steady decrease in body mass was expected (Fig. 3c). The long period of fasting may be associated with the failure to regenerate a limb in one animal of each of the anesthetized and unanaesthetized control group (Fig. 4e) as observed previously in fasted Iberian ribbed newts (*Pleurodeles waltl*)^24^. Thus, although we can confirm hypothesis 4, that unanesthetized and fasting axolotls can perform heart regeneration in both hyposmotic and isosmotic housing medium, this may not always be the case with limb regeneration. A revised hypothesis could be put forward that fasting leads to regenerative failure in a subset of animals. Further studies with larger sample sizes would be required to determine the mechanistic basis of why this occurs only in some animals e.g. potential links to the initial status of adipose tissue deposits and liver glycogen. While this study has established the axolotl’s ability to perform heart and limb regeneration in a comatose state on a gross anatomical level, future experiments should focus in more detail on potential differences in tissue composition e.g. extracellular matrix components and for longer experiments on bone formation and mineralization.

By controlling osmolarity of the housing medium, and administering propofol in a continuous low dose, we could demonstrate that continuous anesthesia for 60 days is possible in the axolotl with a 75% survival rate (Fig. 3), and continuous anesthesia is permissible and does not affect the rate of either heart and limb regeneration compared to unanesthetized control animals (Fig. 4), thus confirming hypothesis 5.

The infusion rate of propofol was gradually adjusted depending on responsiveness from anesthetized animals. We aimed to ensure sufficient depth of anesthesia to keep animals immobile while avoiding propofol overdosage. The necessary infusion rate eventually stabilized at approximately 25% of the initially required infusion rate (Fig. 3b). The clearance of propofol is dependent on both metabolism and distribution to peripheral tissues. Once peripheral compartments have become saturated, the distributional component of clearance decreases^25^, thus resulting in the need for a lower propofol infusion rate to maintain the same level of anesthesia. The need to administer additional bolus doses of propofol to certain animals several times weekly points to individual variation in propofol metabolism despite axolotls being age-matched and of similar size. Combined, this indicates a possible explanation for the two premature deaths in the non-injured anesthetized control group at day 9 post anesthesia. In humans, several cases of propofol-related deaths have been reported and in the majority of cases blood levels of propofol were within the therapeutic range indicating that the cause of death was likely due to respiratory depression with subsequent hypoxia associated with propofol use^26^. Insufficient aeration in the water could have contributed to this potential state of hypoxia leading to death. For the third animal that died at day 56 (anesthetized heart regeneration group), the cause of death was almost certainly lack of oxygen due to a malfunction in the air pump setup.

Regulating long-term anesthesia based on depth of anesthesia rather than using a constant dose over time most likely increases chances of survival. On the other hand, it makes it difficult to untangle the effect of the propofol dose on global parameters such as oxygen consumption rate. We initially, set out to test if heart and limb regeneration affected oxygen consumption by taking advantage of the fact that potential behavioral changes caused by injury would become irrelevant under anesthesia. However, the loss of two uninjured anesthetized control animals made statistical comparisons of oxygen consumption rate between regenerating and healthy animals invalid, thus we failed to test hypothesis 6. However, a comparison of infusion rate of propofol and oxygen consumption rate (Figs. 3b, 5a), indicates that propofol dose generally affects oxygen consumption rate with anesthetized baseline animals in a high concentration propofol bath having a significantly lower oxygen consumption rate than at unanesthetized baseline. Likewise, oxygen consumption was lower at a high dosage of propofol compared to later in the experiment when the same depth of anesthesia could be maintained with a much lower propofol dose. To unravel the link between oxygen consumption and regenerative events and test hypothesis 6 in future experiments would require a larger number of included animals and establishing stable anesthesia before the induction of injury.

Creatinine levels in plasma measured at baseline were below the detection limit. Throughout the experiment, creatinine levels in all groups were found to be lower than the baseline values of 3.1 ± 1.9 mg/dl previously reported for healthy axolotls^27^. This may be a result of the prolonged fasting in the present experiment. A comparison of plasma creatinine levels for animals undertaking limb regeneration indicates that long-term anesthesia does not lead to increases in plasma creatinine levels during limb regeneration (Fig. 5b). Comparatively, plasma creatinine levels tended to increase, though non-significantly, for both anesthetized and unanesthetized axolotls undergoing cardiac regeneration suggesting this to be an effect of cardiac injury rather than a result of long-term propofol exposure^28^ (Fig. 5b).

Propofol cannot with certainty be excluded as the cause of the skin lesions observed after approximately 3 weeks of continuous anesthesia. As indicated by Fig. 5d, the propofol doses used in this study may be associated with some cell death even at low concentrations. However, the concentrations used in this study were 50–250 folds lower than the ones showing signs of marked cytotoxic effects to skin samples. Lesions observed in vivo are therefore likely not the result of propofol toxicity, but potentially associated with immobility similarly to pressure sores in humans. Pressure sores are injuries to skin or soft tissue that form due to prolonged pressure exerted over specific areas of the body. Despite daily checks and rotation of axolotls, the marks shown in Fig. 5c are consistent with the etiology of pressure sores caused by decreased mobility^29^.

In studies involving recovery following long-term anesthesia, neurological effects may be of importance. The limited complexity of axolotl behavior (from an anthropocentric viewpoint) renders it difficult to measure neurological changes other than simple behavior such as willingness to eat and motion behavior. We found that feeding behavior and motion behavior estimated as percentage of active time was unchanged from baseline level at 17 days of recovery, following a period of limited activity (Fig. 5e). This may indicate some early neurological effect of prolonged anesthesia that is either alleviated as residual propofol is gradually washed out or compensated for by neural regeneration^5,30^.

In conclusion, this study shows that axolotls can be kept anesthetized for a period of at least 60 days using propofol as the anesthetic. Long-term anesthesia did not affect the axolotl’s ability to regenerate on the gross anatomical level as demonstrated by the regrowth of amputated limbs and regeneration following cryo-induced cardiac injury. Long-term anesthesia of this scale paves the way for new types of experiments spanning several weeks, such as imaging of live cell migration and scar modulation during regeneration, following the activity of immune cells during regeneration or tracing the metabolism of radiolabeled metabolites in a regenerative species such as the axolotl uncoupled from general behavior. It must be noted, however, that the present study was performed using adult axolotls and piloting would be required to test the applicability of the presented methods in juvenile axolotls often used in limb and tail regeneration studies that may be more sensitive to anesthesia and prolonged fasting. Finally, the described anesthesia method may allow for more severe injury models as the axolotls are not required to awaken after intervention, but can rather perform tissue regeneration in a comatose state.

## Methods

### Study design

The study was designed as three pilot experiments leading to and informing the final study setup. The experimental unit was considered as an individual axolotl and a total of 45 axolotls were included in the study. For pilot experiments there was no prior information to inform sample size, thus the number of animals included was based on availability although with a minimum of three animals per group. Animals that died during experiments were excluded for subsequent analyses based on an a priori decision. Animals were randomized to groups based on an initial random picking. It was not possible to blind the experiments due to the obviousness of animals being anesthetized or unanesthetized. Measurements on individual animals were performed in variable order to reduce potential confounding effects and learning bias.

First, a pilot study (Pilot 1) was conducted to measure waste production, propofol consumption and potential adverse effects of continued propofol anesthesia using conventional hyposmotic tap water housing medium. In this pilot study, six axolotls underwent continued anesthesia for up to 12 days until adverse effects such as body mass increase and swelling were prominent. In the second pilot study (Pilot 2) a total of nine animals were separated into three groups (three animals per group) experiencing increasingly more isosmotic housing medium (tap water, 100% Holtfreter’s solution, axolotl Ringer’s solution) during anesthesia to investigate if this could alleviate problems with swelling. In the third pilot experiment (Pilot 3) it was tested on 12 animals (four animals in three groups) whether conscious axolotls housed in an isosmotic medium (axolotl Ringer’s solution) with and without feeding were able to regenerate the heart following cryoinjury to the myocardium at a similar rate as fasting animals housed in conventional tap water. Finally, after establishing that axolotls can be maintained in propofol anesthesia without swelling for extended periods of time using an isosmotic housing medium and that conscious axolotls housed in an isosmotic medium without feeding can perform heart regeneration, we sought to test whether continuously anesthetized axolotls could perform heart regeneration and/or limb regeneration. We used echocardiography data from Pilot 3 on the effect size regarding the fraction of the non-contracting portion of the ventricle at 4 and 60 days post injury to the heart, respectively, to inform group size (power analysis, α = 0.05, β = 0.2) yielding a minimum group size of three animals to test for cardiac recovery. To take into account that some loss of animals during long term anesthesia could be expected, we decided to increase groups sizes of anesthetized groups to four animals while keeping group sizes of unanesthetized animals to three animals to balance out the ethical desire of reducing the number of used animals while still having a high change for a sufficient group size at the end of the experiment.

### Animals and housing

All used axolotls were obtained from a commercial breeder. All animals were healthy and they were not used for other purposes before experimental start. Prior to experimental procedures animals were fed with axolotl pellets (Axobalance, Aquaterratec) three times weekly with a consistent ingestion between animals. For Pilot 1, six adult axolotls (body mass (BM) ± standard deviation (STD): 63.2 ± 17.4 g) of mixed sexes and color variants were used. For Pilot 2, nine adult axolotls (BM ± STD: 15.0 ± 3.1 g) of mixed sexes and color variants were used. For Pilot 3, twelve adult axolotl (BM ± STD: 45.6 ± 4.8 g) of mixed sexes and color variants were used. In the final experiment of heart and limb regeneration during anesthesia a total of 18 adult, age-matched axolotls (BM ± STD: 20.4 g ± 3.4 g) of mixed sexes and color variants were used. Unanesthetized animals were housed in individual crates at 20 °C on a 12 h light, 12 h dark cycle with weekly water changes. All animals in the final experiment were gradually acclimated to axolotl adjusted amphibian Ringer’s solution. Animals were fasted for the duration of the experiment as well as 14 days prior to start of anesthesia to avoid gastrointestinal complications. Animals were divided into five groups with n = 4 axolotls in each of the anesthesia groups (Anest. control, Anest. limb reg. and Anest. heart reg.) and n = 3 in each of the unanesthetized control groups (Unanest. limb reg. and Unanest. heart reg.). The procedures carried out in this study were in accordance with the national Danish legislation for care and use of laboratory animals. All experiments and housing conditions were approved by the Danish National Animal Experiments Inspectorate (protocol # 2020-15-0201-00688). Humane endpoint was defined as adverse swelling that could not be reduced within the experimental setup. The reporting in the manuscript follows the recommendations in the ARRIVE guidelines.

### Propofol anesthesia

In Pilot 1 all anesthetized animals were housed together in a large 78 × 56 cm^2^ crate containing 10 l of tap water (groundwater, 15°dH, no residual chlorine or chloramines) and the propofol anesthetic. In the other experiments, anesthetized animals were housed individually in 18 × 20 cm crates containing 1 l of either tap water, 100% Holtfreter’s solution (59.21 mM NaCl, 0.67 mM KCl, 0.90 mM CaCl_2_, 2.38 mM NaHCO_3_, total osmolarity = 127.22 mOsm/l) or axolotl adjusted Ringer’s solution (98.38 mM NaCl, 1.74 mM KCl, 1.17 mM CaCl_2_, 2.02 mM NaHCO_3_, total osmolarity = 208 mOsm/l). Propofol (Propofol B, Braun, Melsungen, Germany) in a 10 mg/ml concentration was continuously infused into the housing medium using an infusion pump starting at a rate of 0.109 mg/l/h. The propofol infusion rate was adjusted according to the depth of anesthesia, which was assessed by gently lifting and turning axolotls unto their back to check for a response. If gill movement was observed, additional propofol was administered as a bolus directly into the water. After the weekly water change, animals were kept in propofol free solution for 2–3 h before administering a bolus of 100 μl (1 mg) propofol to each crate. This procedure was performed to allow some wash out of accumulated propofol in the tissue, but without compromising anesthesia.

### Cryo-induced myocardial infarction

Animals were anesthetized and provided analgesia by immersion in benzocaine (200 mg/l solubilized in acetone) in order to perform surgical interventions. Benzocaine was used for surgical anesthesia as propofol is not considered analgesic^31^. Cryoinjury was induced as described by Dittrich and Lauridsen^32^. Briefly, animals were placed in a supine position and the ventricle was exposed by a small incision in the skin and pericardium. A liquid nitrogen cooled probe was applied to the ventricle for 10 s before being gently detached by flushing with sterile amphibian Ringer’s with 0.1× antibiotic/antimycotic solution (Sigma, cat. no. A5955). The pericardium was stitched together with 1–2 sutures (8-0 suture, non-absorbable), cartilage plates put back into place, and skin was stitched together with 3–5 stitches (7-0 suture, non-absorbable). The entire animal was covered in wet paper towels and placed on ice for 1.5 h to allow for the rapid initiation of epidermal regeneration before the animal was transferred back into the housing crate.

### Limb amputation

Prior to amputation, animals were anesthetized and provided analgesia by immersion in benzocaine (200 mg/l) for 30 min. The right front leg was amputated approximately mid humerus. After 1 min, skin contraction was complete and the protruding bone was trimmed flush with the wound surface. The animal was then placed on ice for 1.5 h before being transferred into a clean housing crate.

### Magnetic resonance imaging

MRI was performed to measure fluid accumulation in tissues during anesthesia in the pilot 1 experiment using a clinical 3 T Siemens Magnetom Skyra system. Animals were scanned immediately following anesthesia and again after 3 days in anesthesia. For each scan, axolotls were positioned head first in a prone position. A T2-weighted spin-echo sequence was acquired with the following parameters: repetition time = 1030 ms, echo time = 141 ms, excitation flip angle of 120°, four averages and an isotropic image resolution of 0.45 mm.

### Echocardiography analysis of non-contraction fraction

Non-contraction fraction, i.e. the size of the injured ventricle relative to the healthy contracting portion of the ventricle, was analyzed using a Vevo2100 ultrasound imaging system (FUJIFILM/Visualsonics Inc., Canada) with a high frequency transducer (VisualSonics MS550S, 40 MHz center frequency). Animals were placed in a supine position in a metal tray containing enough anesthesia solution to cover the animal. The transducer position was adjusted to capture ventricular cross-sections in maximal long and short axis as well as maximum infarction area for both planes. All measurements were performed using the Vevo 2100 software (Vevo LAB 3.2.6) and averaged over 3 cardiac cycles. Non-contraction fraction (NCF) was calculated after measuring area (A) of the non-contraction zone in long and short axis and area of the ventricle at full diastole in long and short axis using the formula:

$$NCF=\frac{{A}_{NC, long axis}+{A}_{NC, short axis}}{{A}_{Diastolic, long axis}+{A}_{Diastolic, short axis}}\times 100\%$$

### Respirometry

Closed respirometry was performed using 1.1 l gas impermeable glass boxes completely filled with temperate Ringer’s solution and an O_2_-microsensor (Unisense, Denmark) to measure the oxygen saturation in the water. The O_2_-microsensor was calibrated using aerated Ringer’s (20.95% O_2_) and oxygen saturated Ringer’s solution (100% O_2_). Start oxygen saturation was measured and the container was sealed with the resting axolotl inside. After 2 h, end oxygen saturation was measured in order to calculate the amount of oxygen consumed during the time period. Temperature and water mass in the container was measured and adjusted for as well as daily barometric pressure and steam pressure. Awake and anesthetized respirometry was conducted prior to surgical intervention and then repeated throughout the main experiment at day 4, 14, 35 and 60 post cardiac injury or leg amputation.

### Blood sampling and creatinine measurement

Blood samples were collected on Pilot 1 animals to measure plasma osmolarity and again in the main long-term anesthesia experiment to monitor plasma creatinine as an indicator for kidney function. Approximately 50 μl of blood was collected from a gill artery using a heparinized 0.5 ml syringe with a 30 gauge needle. Samples were centrifuged at 2000*g* for 5 min and plasma was transferred to a clean cryotube and snap-frozen in liquid nitrogen. Creatinine in plasma was measured using the QuantiChrom Creatinine Assay from BioAssay Systems (cat. no. DICT-500) as described in the manufacturer’s protocol. We attempted to measure blood glucose levels using a Freestyle Precision Neo Blood glucose apparatus, however, at 4 days post injury, i.e. after 18 days of fasting, glucose levels were below the detection limit of the apparatus.

### Immunohistochemistry and histological quantification of infarction fraction

For immunohistochemical analysis, cryosections were prepared by embedding harvested hearts in M-FREEZE Cryoembedding media (Sigma, cat. no. 103693) followed by snap-freezing on a plastic cradle floating on liquid nitrogen. Hearts were cryo-sectioned at a 10 μm slice thickness and post-fixed with 4% paraformaldehyde and permeabilized in 0.25% Triton X-100 in 70% phosphate buffered saline for 15 min. Sections were blocked with blocking solution (3% bovine serum albumin, 5% goat serum, 0.1% Triton X-100 in 70% phosphate buffered saline) for 3 h and incubated overnight at 4 °C with the primary antibody (anti α-actinin (Sigma, cat. no. A7811)). Sections were incubated with secondary antibody (Goat anti Mouse IgG1 Alexa Fluor 647 (Invitrogen, cat. no A21240)) for 3 h at room temperature. Sections were subsequently co-stained with FITC-conjugated wheat germ agglutinin (ThermoFisher, cat. no. W11261) and incubated for 1 h at room temperature before mounting using Prolong Diamond Antifade Mounting Media with DAPI (Invitrogen, cat. no. P36965). Sections were scanned using an Olympus VS120 slide scanner and analyzed using QuPath. Quantitative histology was performed by identifying the sections with the largest infarctions and then measure infarction size (green signal) relative to the size of the ventricle (red myocardium signal) to yield the infarction fraction. This value was averaged over three sections for each heart.

### Blastema imaging and limb regeneration assessment

Amputated limbs were photographed to track the regenerative progress. These images were used as a comparison of the regenerative ability in unanesthetized and anesthetized animals during limb regeneration. The regrowth of the amputated limb was measured as the length to diameter ratio using ImageJ 1.50e.

### Propofol toxicity

Dermal tissue samples from the abdominal area of non-propofol treated axolotls sacrificed for other purposes were excised and incubated for 1 h in different propofol concentrations. Skin samples were incubated with either 0, 0.0002, 0.1, or 0.5 mg/ml propofol in 70% minimum essential medium at room temperature with mild agitation (200 rpm). Following incubation, medium was analyzed for cytotoxicity using the CyQUANT LDH Cytotoxicity Assay Kit (Invitrogen, cat. no. C20300). The lactate dehydrogenase release was normalized to the weight of tissue in each well and the absorbance from the 0 mg/ml well was subtracted to account for the cell death simply caused by excision of the sample.

### Behavioral assessment

To asses potential behavioral changes caused by propofol anesthesia, axolotl crates containing individual animals were placed on an even blue background with a high level of contrast compared to both white and dark animals. A time lapse wirelessly operated camera (GroPro Hero 4, GoPro, Inc., San Mateo, CA, USA) was placed above the animals which were left to rest over night. On the following day (at the same time during the day for each assessment) the time lapse camera was remotely operated and the undisturbed animals were filmed for 90 min. Video material was manually analyzed for the percentage of the recording that individual animals were actively moving around. Originally (at baseline before anesthesia), only anesthetized control animals i.e. receiving no cardiac injury or limb amputation were included in the behavioral assessment to rule out potential effects of surgery. However, the loss of two animals in this group early in the experiment lead us to include also the animals that had performed limb amputation during anesthesia in the behavioral assessment at day 7 and day 17 post recovery from anesthesia.

### Statistical analysis

Data is shown throughout as mean values ± standard deviations. Analyses of significant differences between two groups were performed in Microsoft Excel using a two-tailed Student’s t-test (paired or unpaired), and for more than two groups using ANOVA. For repeated measures, one-way or two-way ANOVA with repeated measures was performed with Tukey’s honest significance test for post hoc test of significant differences between groups. We considered p values less than 0.05 to signify statistical significance and throughout figures we use the terminology *p < 0.05, **p < 0.01, ***p < 0.001, ns: not significant.

## Data Availability

The raw data of the study is available upon reasonable request from the corresponding author.
